# Genomics for next‐generation wheat breeding

**DOI:** 10.1002/tpg2.70263

**Published:** 2026-06-04

**Authors:** Rajeev K. Varshney, Vanika Garg, Manuel Spannagl, Susanne Dreisigacker

**Affiliations:** ^1^ Centre for Crop and Food Innovation (CCFI), WA State Agricultural Biotechnology Centre Murdoch University Murdoch Western Australia Australia; ^2^ Helmholtz Munich German Research Center for Environmental Health Neuherberg Germany; ^3^ International Maize and Wheat Improvement Center (CIMMYT) Texcoco Mexico

## 1 INTRODUCTION

Meeting global food demand in the face of climate change and resource scarcity presents agriculture's most pressing challenge (Ray et al., 2012; Tilman et al., 2011). Wheat, the world's most widely cultivated cereal crop, is at the center of this challenge. With over 750 million tonnes produced annually and consumed by approximately 1.5 billion people worldwide, wheat underpins food security across diverse geographies and economic contexts (Shewry & Hey, 2015). Yet, recent trends in wheat production are concerning. After decades of consistent productivity growth driven by improved varieties and farming practices, yield growth rates have decelerated dramatically in major producing regions since the 1990s (Grassini et al., 2013). In many countries, current yields have plateaued or begun to decline relative to historical trends, suggesting that conventional approaches to productivity improvement are reaching their limits (Fischer & Edmeades, 2010).

This deceleration coincides with mounting environmental pressures, including water scarcity, soil degradation, and intensifying pest and disease pressures (Lobell et al., 2011), and is exacerbated by structural constraints within wheat's own genetic architecture. More than a century of selection for yield has progressively narrowed the genetic foundation of elite wheat germplasm, reducing the additive variation available for directional selection and adaptive capacity for novel environmental stresses (Cheng et al., 2024). Wheat's polyploid nature further complicates breeding by creating complex segregation patterns and linkage relationships that impede trait combination (Gill & Friebe, 2002).

Beyond these genetic constraints, contemporary breeding faces inherent trade‐offs between competing objectives. Increasing grain protein content required for breadmaking quality often reduces yield potential (Zörb et al., 2018). Enhancing disease resistance through introgression from wild relatives frequently introduces linkage drag, negating gains in other traits (Kale et al., 2025). Reducing dependence on nitrogen (N) fertilizer, essential for environmental sustainability, conflicts with maintaining the high protein concentrations demanded by grain processors. These interconnected challenges are too complex to be resolved through incremental improvements of existing breeding methods.

Genomic technologies offer novel routes to overcome these breeding constraints (Shewry & Hey, 2015). Complete wheat genome sequences provide reference frameworks for identifying and characterizing genetic variation at unprecedented resolution (International Wheat Genome Sequencing Consortium, 2018). Pangenomic resources catalog structural variation and allelic diversity across diverse wheat populations, revealing beneficial alleles absent from elite breeding lines (Jiao et al., 2025). Genome‐wide association studies (GWAS) enable rapid identification of loci underlying complex traits across diverse germplasm (Gudi et al., 2025). Genomic selection accelerates breeding cycles by enabling selection based on DNA marker‐derived genomic estimated breeding values (GEBVs) in early breeding generations rather than waiting for extensive phenotypic characterization across multienvironmental trials, reducing time from cross to released variety by several years (Meuwissen et al., 2001). Precise gene editing technologies permit the introduction of favorable alleles from wild relatives without accumulating unfavorable linked variation (Svitashev et al., 2016). When these tools are integrated with high‐throughput phenotyping platforms, advanced statistical methods, and international data sharing networks, they create opportunities to systematically interrogate trait architecture, understand genotype‐by‐environment interactions, and develop more effective breeding strategies tailored to specific production environments and end‐use requirements.

**FIGURE 1:**
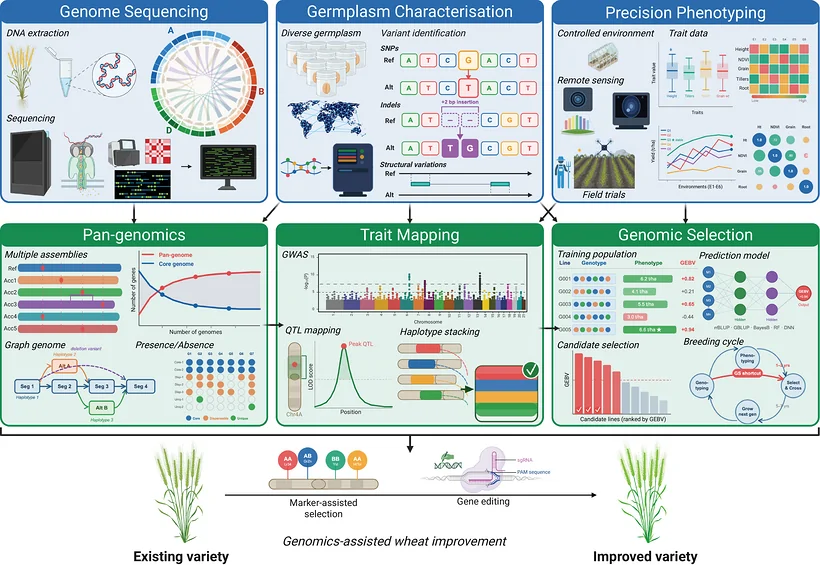
Integrated genomics‐assisted wheat improvement. Systematic wheat improvement integrates genome sequencing, germplasm characterization, and precision phenotyping to generate comprehensive datasets on genetic and trait variation across multiple environments. These discovery datasets feed into pangenomic analysis, which reveals structural variation and allelic diversity, expanding the accessible genetic space for breeding. Integration of pangenomic insights with genome‐wide association studies (GWAS) and quantitative trait loci (QTL) mapping enables the identification of genomic regions controlling agronomic traits and defines optimal haplotype combinations for trait stacking, facilitating the pyramiding of favorable alleles for stress resilience, yield stability, and nutritional quality. Genomic selection builds predictive models from datasets to estimate genomic estimated breeding values (GEBVs), enabling marker‐based selection of elite breeding parents at juvenile stages and dramatically accelerating breeding cycles compared to conventional approaches. These genomic discoveries translate into practical breeding strategies through marker‐assisted selection for rapid trait introgression and precision gene editing for targeted modification of regulatory regions, which, when integrated with speed breeding, doubled haploid production, and field evaluation, deliver improved varieties. The research papers in this special issue exemplify and advance key components of this integrated workflow.

The 3rd International Wheat Congress (IWC), held in September 2024 in Perth, Western Australia, and hosted by Murdoch University's Centre for Crop and Food Innovation and the WA State Agricultural Biotechnology Centre, brought together researchers and breeders to evaluate progress in applying genomic approaches to wheat improvement. This special issue of *The Plant Genome* presents 12 papers, including one review article from IWC participants, that collectively exemplify how genomic and physiological insights are being translated into practical crop advancement. These contributions address trait discovery and mapping for grain nutrition, environmental stress tolerance, yield components, and disease resistance; large‐scale characterization of breeding germplasm; and integrated breeding strategies. The papers demonstrate both the power of genomic approaches and the complexity of translating genomic discoveries into agronomically superior varieties (Figure 1). This editorial synthesizes key insights from these contributions and examines how genomic tools are reshaping both the fundamental questions wheat researchers ask and the practical strategies breeders employ to develop resilient, productive, high‐quality varieties for diverse production systems.

## 2 GENOMIC DISSECTION OF ABIOTIC STRESS TOLERANCE

As climate variability intensifies, understanding the genetic and molecular foundations of abiotic stress tolerance has become central to sustaining wheat productivity. Among the most pressing of these stresses is heat, which severely impairs wheat development, particularly at the seedling stage. Gudi et al. (2025) characterized this vulnerability through an evaluation of a diverse panel of landraces and cultivars under controlled conditions at 23°C and 36°C. Heat stress uniformly suppressed seedling development, though with differential genotypic susceptibility: root elongation was most severely compromised (averaging 85.6% reduction), while coleoptile length showed relative resilience (15.4% reduction). Screening identified six genotypes exhibiting exceptional heat tolerance (PI 366905, Kzyl Sark, Rang, Perico S, Bohr Gamh, PI 620689) and six with marked susceptibility (CItr 17470, CItr 13270, Coeruleum, Shashi, Hallany, Currawa), enabling the identification of genomic variation underlying this trait. GWAS using >300,000 single nucleotide polymorphisms (SNPs) revealed 23 marker–trait associations, with 16 specifically linked to seedling performance under heat stress. Functional annotation of differentially expressed genes identified 35 loci responding to heat, of which 13 emerged as high‐confidence candidates: protein kinases, basic‐leucine zipper transcription factors, UDP‐glucosyltransferases, pyrophosphate‐energized proton pumps, and fatty acid hydroxylases. These genes represent molecular entry points for developing heat‐resilient cultivars through favorable allele selection and marker‐assisted breeding.

Hosseini Pouya et al. (2025) examined a complementary stress response system: calcium signaling under drought. GWAS identified 23 *calcineurin B‐like* (*CBL*) genes in durum wheat organized into four evolutionary clusters. Regulatory element analysis revealed promoter sequences responsive to abscisic acid (ABA) signaling as well as direct stress perception, indicating that *CBL* genes coordinate multiple defense pathways. Under drought conditions and exogenous ABA application, expression patterns varied substantially across tissues. Two genes, *TtCBL2* and *TtCBL10*, demonstrated strong drought‐responsive activity specifically in root tissues, making them particularly valuable for improving belowground drought tolerance. *TtCBL19* emerged as a regulatory hub coordinating both ABA‐dependent and independent stress responses, suggesting broader utility in cross‐tolerance mechanisms. The integration of computational pathway analysis with experimental expression profiling provides a rational framework for identifying breeding targets, moving beyond phenotypic screening to functional gene prioritization.

Translating drought‐responsive gene networks into improved yield under water‐limited conditions requires connecting molecular mechanisms to agronomic performance. Caguiat et al. (2026) pursued this connection by developing near‐isogenic lines targeting a QTL for 1000‐grain weight on chromosome 3B under drought stress. Using heterogeneous inbred family methods and fast‐generation cycling, near‐isogenic lines were genotyped with a 90K SNP array, narrowing the target QTL region and identifying four SNP markers linked to six candidate genes. These genes encode proteins with direct relevance to drought stress responses: beta‐glucuronosyltransferase, ABC transporter, transmembrane protein, F‐box protein, cytochrome P450, and alanine‐tRNA ligase. Expression analysis revealed elevated expression of all six candidate genes in root and grain tissues specifically under drought stress. Pathway analysis indicated their involvement in ubiquitin‐mediated proteolysis, metabolic pathways, and biosynthesis of N‐glycans, steroids, and secondary metabolites, which are the molecular processes fundamentally linking stress physiology to grain development. This work demonstrates how drought‐responsive signaling networks identified at the molecular level translate into quantitative control of yield traits, providing both SNP markers and candidate genes for breeding high‐yielding, drought‐resilient cultivars.

Whiting et al. (2025) investigated environmental resilience from a geographic perspective, examining winter survival in Canadian wheat production systems. While traditional cold tolerance markers, *Vrn‐A1* and *Fr‐A2*, are essentially fixed throughout elite Canadian germplasm, rendering conventional marker‐based selection ineffective, substantial variation in winter survival persists among genotypes carrying these alleles. Evaluation of 321 genotypes fixed for beneficial *Vrn‐A1* and *Fr‐A2* alleles across three environments followed by GWAS identified three QTLs controlling winter survival: one previously mapped locus on chromosome 5A and two novel regions on chromosomes 5D and 7B. Further, analysis of these QTL intervals revealed an unexpected finding: genes involved in diverse biotic and abiotic stress responses, not solely cold tolerance were enriched in regions associated with winter survival. This suggests that winterkill in this region results from multiple intersecting stressors rather than a single temperature threshold. Consequently, screening individual stress components under controlled conditions may prove more tractable than field validation of rare catastrophic winter events.

Together, these studies demonstrate that environmental resilience is not controlled by isolated stress–response modules but emerges from coordinated action across multiple genetic networks and signaling pathways. Strategic application of in silico analysis to prioritize functional genes, combined with targeted physiological characterization, provides a more efficient breeding strategy than traditional single‐trait empirical approaches. This integration of computational and experimental evidence allows breeders to identify alleles with genuine functional relevance across stress contexts.

## 3 YIELD ARCHITECTURE AND REPRODUCTIVE DEVELOPMENT

Grain yield depends on the coordinated development of multiple components. Wang et al. (2025) fine‐mapped a gene controlling tiller initiation through analysis of the *ot2* wheat mutant, which exhibits a dramatic 91% reduction in tiller number compared to the wild type. The mutant shows inhibited tiller bud differentiation from the two‐ to three‐leaf stage onward, a critical developmental window. Using bulked segregant analysis combined with exon sequencing, the *Taot2* locus was mapped to chromosome 1BL, and 14 Kompetitive Allele‐Specific PCR (KASP) markers were developed to narrow the region to a 2.22‐Mb interval encompassing 39 high‐confidence genes. Transcriptomic analysis and functional investigation identified *TraesCS1B03G1126300* as the candidate gene, encoding an auxin‐responsive protein of the auxin/indole‐3‐acetic acid family. This identification reveals how auxin signaling regulates bud differentiation early in plant development. By understanding this developmental switch, breeders can now modulate plant architecture across different production systems by adjusting tiller number in response to specific agronomic contexts and resource availability.

Rohde et al. (2025) synthesized knowledge about reproductive development in wheat to enable hybrid wheat production. Critical research priorities were identified for advancing hybrid systems, including floral morphology optimization, pollination efficiency, male sterility mechanisms, and reproductive trait stability under environmental stress. Together, these contributions reveal that yield improvement requires not simply identifying yield components but understanding the developmental and physiological processes that govern their formation. This mechanistic understanding transforms yield architecture from an empirical selection target into a rational design problem amenable to targeted breeding and genomic approaches.

## 4 NUTRITIONAL TRAITS AND YIELD STABILITY

Micronutrient deficiency remains a pervasive global health challenge, with zinc deficiency alone affecting approximately 17% of the world's population, disproportionately impacting regions where wheat‐based diets dominate. Despite this scale of impact, breeding for improved grain nutrition has historically taken a back seat to yield maximization, leaving substantial nutritional gains untapped. Zeng et al. (2025) address this gap by mining wild emmer wheat (*Triticum dicoccoides*), a rich but underexploited reservoir of genetic variation that has contributed substantially to modern bread wheat. Through GWAS analysis in tetraploid wheat populations, a stable QTL on chromosome 4AS was identified that increases grain zinc concentration without penalty to yield components, an important distinction given the historical inverse correlation between yield and grain mineral content. Validated KASP markers were developed alongside the identification of a candidate gene involved in zinc transport, providing breeders with concrete tools to introgress this trait into elite varieties. This work demonstrates that the systematic use of wild genetic resources can simultaneously address nutritional objectives while maintaining agronomic performance, supporting global biofortification breeding programs, such as HarvestPlus, and contributing to broader strategies to tackle hidden hunger through improved staple crops.

## 5 GENETIC ARCHITECTURE OF MULTIRUST RESISTANCE

Wheat rust remains a persistent and evolving threat to global wheat production. Stripe rust, caused by *Puccinia striiformis* f. sp. *tritici* (*Pst*), exemplifies the complexity of durable resistance breeding. As Jan et al. (2026) comprehensively review, the *Pst* pathogen thrives in cool, humid environments and exhibits remarkable evolutionary agility, regularly producing virulent pathotypes that overcome deployed resistance genes. The recurrent breakdown of single resistance genes reflects the pathogen's genomic plasticity and capacity for rapid adaptation. This review synthesizes current understanding of stripe rust resistance, spanning pathogen biology, multilayered host immune signaling (pattern recognition receptors, major resistance genes, and QTL), and downstream defense cascades. Multi‐omics studies have revealed biphasic defense responses and regulatory networks distinguishing resistant and susceptible genotypes, highlighting the complexity of host–pathogen interactions. Emerging roles of transcription factors, epigenetic regulation, and gene regulatory networks underscore that durable resistance requires understanding not just individual resistance genes but the broader genetic and regulatory architecture controlling defense.

Given this complexity, Jan et al. (2026) emphasize that effective breeding for durable stripe rust resistance must integrate multiple complementary strategies: classical selection, QTL mapping, GWAS, marker‐assisted selection, genomic selection, and genome editing. Advances in genome sequencing, pan‐genomics, and allele mining are accelerating the discovery of novel resistance sources and expanding the understanding of wheat genetic diversity; however, connecting these genomic discoveries to practical breeding remains challenging.

Joukhadar et al. (2025) demonstrated one powerful approach to bridge this gap by integrating diverse populations and multienvironment field evaluations into a comprehensive meta‐genome‐wide association study (metaGWAS). Their study encompassed five globally distributed populations phenotypically evaluated across 16, 13, and 19 field experiments for leaf, stem, and stripe rust resistance, respectively, generating 12,694, 10,725, and 16,281 total phenotypic observations. Despite moderate heritability estimates, the metaGWAS approach substantially enhanced QTL detection stability across environments. The analysis identified 19 QTLs for leaf rust resistance, 17 for stem rust resistance, and five for stripe rust resistance. Critically, six QTLs conferring resistance to multiple rust types were identified, suggesting pleiotropic loci valuable for durable, multipathogen resistance breeding.

Complementary to this work, Sivakumar et al. (2026) conducted a detailed haplotype analysis of rust resistance traits, identified multirust resistance haplotype combinations, and developed validated markers linked to elite accessions. These parallel approaches, combining metaGWAS discovery and haplotype‐based marker development, together with the genomics and multi‐omics framework outlined by Jan et al. (2026), illustrate how multiple complementary strategies can triangulate toward practical breeding solutions for durable, multipathogen resistance. Such integrated approaches are particularly critical given ongoing pathogen evolution and the anticipated impacts of climate change on rust epidemic dynamics, underscoring the need for continuous innovation in resistance breeding and sustainable wheat production.

## 6 SCALING GENOMICS TO GLOBAL BREEDING PROGRAMS

Large‐scale genotyping initiatives are advancing breeding strategies through systematic germplasm characterization. Shrestha et al. (2025) genotyped 130,247 spring bread wheat lines bred by the International Maize and Wheat Improvement Center (CIMMYT) breeding program (2013–2023), constructing 636 genotyping‐by‐sequencing libraries with 96‐384‐plex multiplexing to generate 30.7 terabases of sequence data. An optimized TASSEL pipeline identified 24,125 high‐quality SNPs across all 21 chromosomes. Population genetic clustering of 444 selected lines within 10 pedigrees validated genotyping accuracy. Nucleotide diversity and minor allele frequency analyses revealed significantly reduced genetic variation in pericentromeric regions across all chromosomes. This pattern was confirmed through comparison with winter wheat accessions, indicating fixation of large centromeric haplotype blocks in elite spring wheat germplasm. Temporal pairwise F_ST_ analyses identified selection signatures consistent with published GWAS for agronomic traits, including grain yield and disease resistance. These findings demonstrate that recombination suppression in pericentromeric regions constrains accessible genetic diversity for breeding. The publicly distributed dataset provides a critical resource for genomic prediction and breeding program optimization at a global scale, enabling inference that individual programs cannot generate independently.

## 7 BREAKING THE YIELD‐PROTEIN TRADE‐OFF

Grain quality and nutritional value are increasingly important considerations for wheat breeding, particularly as global food security intersects with environmental sustainability. Elevated grain protein content (GPC) directly improves end‐use quality and supports nitrogen utilization efficiency, reducing the environmental damage associated with excess fertilizer inputs. However, breeding progress in improving GPC has been constrained by a fundamental trade‐off: a strong negative correlation between grain protein content and grain yield. Recent cultivar development has exacerbated this constraint, as modern elite lines show higher yields but declining protein levels.

Kale et al. (2025) addressed this challenge by analyzing multilocation, multiyear phenotypic data from winter wheat varieties in Scandinavian regions, examining variation in grain protein content, grain yield, and grain protein deviation. GWAS analysis of two independent populations identified significant marker–trait associations for both GPC and grain protein deviation (GPD), with chromosome 2B emerging as a critical locus controlling these traits. A key finding revealed that an introgression from *Triticum timopheevii*, introduced into elite germplasm to confer powdery mildew resistance, coincides with reduced GPC and GPD. This deleterious effect, likely resulting from linkage drag in the introgressed region, demonstrates how beneficial alleles for disease resistance become entangled with unfavorable variants affecting protein accumulation. The authors advocate for using advanced genomic techniques such as CRISPR‐Cas and mutagenesis to break these unfavorable linkages, allowing disease resistance and high protein content to segregate independently.

This example illustrates a critical challenge in modern wheat breeding: managing pleiotropic effects and linked traits to achieve multiple objectives simultaneously. The underlying regulatory networks controlling protein and starch accumulation remain incompletely understood, but chromosome 2B's central role in GPC regulation offers a focal point for further dissection. Identifying the specific genes and regulatory elements controlling this locus could enable targeted improvements in protein content and quality without yield compromise.

## 8 FUTURE PERSPECTIVES

This special issue documents the maturation of genomic tools within wheat breeding programs, while simultaneously revealing the depth of complexity in translating genetic discoveries into improved varieties. The papers assembled here demonstrate that identifying genetic variants and mapping QTL are necessary but insufficient steps; success increasingly depends on integrating these discoveries with physiological understanding, agronomic data, and strategic breeding decisions. Several constraints emerge as immediate priorities for future research. Trait combinations often carry unfavorable linkages that resist conventional breeding timelines, as Kale et al. (2025) illustrate with the T. timopheevii introgression affecting both disease resistance and protein content. Selection for stable performance across variable environments remains challenging when training populations cannot fully capture genotype‐by‐environment interaction. The pace of variety deployment lags disease evolution and climatic shifts, as demonstrated by the recurring breakdown of rust resistance genes documented by Jan et al. (2026). These are coordination challenges spanning genomics, physiology, and breeding logistics rather than failures of genomic resolution alone.

Looking forward, several converging technologies promise to address these constraints systematically. Pan‐genomic resources will increasingly replace single reference genomes, capturing structural variation and rare alleles absent from elite breeding pools. Machine learning frameworks will enable the integration of heterogeneous phenotypic, genotypic, and environmental data, supporting prediction of variety performance across diverse production systems. Speed breeding combined with high‐throughput phenotyping will compress selection cycles, while precision genome editing will permit targeted modification of regulatory regions to fine‐tune trait expression. International data sharing networks, exemplified by the CIMMYT's publicly distributed genotyping dataset (Shrestha et al., 2025), will democratize access to genomic resources that no single program could generate independently.

The overarching challenge is coordinating these advances and translating them into coherent breeding strategies. Success will require sustained dialogue across disciplines between molecular and quantitative geneticists optimizing analytical pipelines, physiologists and pathologists characterizing trait performance, and breeders making field‐level decisions. Climate change, pathogen evolution, and growing food security demands amplify the urgency. The integration demonstrated across this collection provides not only a template for current breeding programs but an imperative for the decade ahead: genomic technology must be paired with biological insight and practical breeding judgment to deliver the resilient, productive, nutritious wheat varieties global agriculture requires.

## AUTHOR CONTRIBUTIONS

**Rajeev K. Varshney**: Conceptualization; writing—original draft; writing—review and editing. **Vanika Garg**: Visualization; writing—original draft; writing—review and editing. **Manuel Spannagl**: Writing—review and editing. **Susanne Dreisigacker**: Writing—review and editing.

## CONFLICT OF INTEREST STATEMENT

Rajeev K. Varshney and Susanne Dreisigacker are members of the Editorial Board of *The Plant Genome*, and Rajeev K. Varshney and Vanika Garg have active collaborations with the Editor‐in‐Chief, Henry Nguyen. In addition, several authors contributing to this special issue have current or past collaborative relationships with one or more of the Guest Editors. These relationships had no influence on the editorial handling, selection of reviewers, or peer‐review process for the manuscripts included in this issue. Appropriate measures were implemented to manage this potential conflict of interest. Rajeev Varshney was not involved in the editorial review process, and editorial decisions were made in line with the standard journal procedures. All submissions underwent the journal's standard peer‐review procedures, which were conducted with full impartiality and in accordance with the journal's policies to maintain the integrity of the review process.
